# Force in robotic thoracic surgery –a one year analysis of DaVinci 5 force feedback

**DOI:** 10.1007/s11701-025-02781-9

**Published:** 2025-09-25

**Authors:** Peter J. Kneuertz, Robert Mostellar, Robert E. Merritt, Elliot L. Servais, Brian Mitzman, Nestor R. Villamizar, Luis F. Tapias, John F. Lazar, Desmond M. D’Souza, Daniel S. Oh, Gretchen P. Jackson

**Affiliations:** 1 Division of Thoracic Surgery, Department of Surgery, The Ohio State University, Wexner Medical Center, 410 W 10Th Ave, Columbus, OH 43210 USA; 2 Intuitive Surgical, Sunnyvale, CA USA; 3 Division of Thoracic Surgery, Lahey Hospital and Medical Center, Burlington, MA USA; 4 Department of Surgery, University of Utah Health, Salt Lake City, UT USA; 5 The DeWitt Daughtry Family Department of Surgery, University of Miami Miller School of Medicine, Miami, FL USA; 6 Division of Thoracic Surgery, Department of Surgery, Mayo Clinic, Rochester, MN USA; 7 Division of Thoracic Surgery, Ascension Saint Thomas Hospital, University of Tennessee Health Science Center, Nashville, TN USA; 8 Division of Thoracic Surgery, City of Hope, Duarte, CA USA; 9 Department of Pediatric Surgery, Vanderbilt University Medical Center, Nashville, TN USA

**Keywords:** Robotic surgery, Thoracic surgery, Instrument forces, Haptic feedback, Da Vinci

## Abstract

Lack of tactile sensation has been a limitation of robotic-assisted thoracic surgery (RATS). The da Vinci 5 System (launched in 2024) integrates force feedback (FFB), a technology that measures instrument-tip forces and relays them to console hand controllers. This study characterizes forces applied during RATS and evaluates the impact of FFB. Da Vinci 5 system data for all RATS procedures using FFB instruments performed in the US between March 29, 2024 and April 30, 2025 were reviewed. Common thoracic surgeries were analyzed, including lung resections, mediastinal, esophageal, and diaphragmatic procedures. Mean instrument-tip forces (Newtons, N) were compared by procedure and instrument type. Forces and time spent above > 6.5 N were compared across FFB settings. Data from 444 procedures by 73 unique surgeons were analyzed. Median forces during lung resections (1.45 N anatomic resection, 1.42 N wedge) were significantly lower compared to mediastinal procedures (1.61 N), esophageal (1.74 N), and diaphragm surgery (1.59 N) (*p* < 0.001). Higher forces were measured with the use of retraction (Cadiere or Fenestrated Bipolar) than dissecting (Maryland) instruments (*p* < 0.001). Median forces decreased progressively with higher FFB settings (off, 1.79 N vs. low, 1.67 N vs. medium, 1.45 N vs. high, 1.32 N, *p* < 0.001). Proportion of duration at force > 6.5 N similarly declined with increased FFB setting (*p* < 0.001). FFB technology is associated with reduced average and peak instrument forces during RATS, particularly at medium and high settings. Retraction instruments experienced the highest forces. Further research is needed to define optimal force thresholds and clinical impact.

## Introduction

Robotic-assisted thoracoscopic surgery (RATS) has reduced the learning curve and broadened applications for minimally invasive thoracic surgery, enabling surgeons to perform more advanced procedures thoracoscopically [1, 2]. RATS has addressed many technical limitations of traditional video-assisted thoracoscopic surgery (VATS) by offering improved dexterity with wristed instruments, motion scaling, tremor filtration, enhanced 3D visualization, relaxed ergonomics, and surgeon control of all instruments and the camera. However, the lack of tactile feedback has remained an important limitation of RATS. The inability of surgeons to feel force exerted in the operative field requires adjustments in retraction and dissection techniques compared to VATS or open surgery [3]. Surgeons develop visual haptics by training their eyes to register and interpret visual cues on how the tissue reacts during surgery, but static forces and forces on tissue under tension are more difficult to register [4, 5].

The da Vinci 5 (dV5) Surgical System (Intuitive Surgical, Sunnyvale, CA) incorporates Force Feedback (FFB) instruments with sensors that measure push and pull forces at instrument tips and transmit them to hand controllers on the robotics console [6]. The system was launched in 2024 in the US. FFB versions of instruments commonly used in thoracic surgery, including the Cadiere Forceps, Fenestrated Bipolar Forceps, Maryland Bipolar dissector, and Needle Driver, became available.

To date, little is known about the forces applied during RATS. The objectives of this study were to characterize the range of robotic instrument forces applied during RATS and to determine the impact of FFB use.

## Methods

We utilized the Intuitive da Vinci robotic system data for the most common types of thoracic surgery procedures, including lung resections, mediastinal procedures, transthoracic esophageal procedures, and diaphragmatic procedures performed on the dV5 surgical system using at least one FFB instrument from system launch on March 29, 2024, to April 30, 2025. Cases with extreme outliers in console time (< 5 min or > 7 h) were excluded, as were observations with FFB utilized < 5 s. The study leveraged protocols for analyses of da Vinci Surgical System and My Intuitive data, which were deemed exempt and approved by the WCG Institutional Review Board (Protocol# ISI 20221) on November 21, 2022.

### Data source and definitions

We analyzed a limited dataset derived from the Intuitive dV5 surgical systems, from which surgeon, patient, and institutional identifiers were removed. For each case, data collected included the console time, type of instruments used, and whether the dual console was engaged. Surgeon experience level was determined by the number of procedures performed on any da Vinci system prior to the case. Instrument tip forces for FFB instruments were recorded as scalar values in Newtons (N), pertaining only to the X, Y, and Z axes at the instrument tip, and do not include grasping forces. Forces were analyzed relative to the feedback sensitivity settings (i.e., off, low, medium, and high), which was chosen by the operating surgeon.

### Statistical analysis

Descriptive statistics were applied to summarize procedure characteristics, dual console setup, and instruments used. Mean instrument tip forces were computed for every discrete instrument and force setting used during a given case and compared using Kruskal–Wallis and post-hoc Mann–Whitney U tests, stratified by procedure type and instrument type. Median forces and duration of peak forces over 6.5 N relative to the overall force application time were compared by FFB setting. Data analysis was performed by RM using Python (version 3.10.12; Python Software Foundation, https://www.python.org) with statistical and visualization packages pandas, numpy, scipy, statsmodels, itertools, matplotlib, and seaborn.

## Results

A total of 444 thoracic procedures were performed on the dV5 with FFB instruments during the study period. Anatomic lung resection was the most commonly performed procedure (58.2%, *n* = 259), followed by wedge resection (21.8%, *n* = 97) and mediastinal surgery (9.7%, *n* = 43). Fewer patients underwent transthoracic esophagectomy or diaphragmatic surgery (Table 1). The procedures were performed by 73 unique surgeons with a median of 592 (IQR = 843) prior da Vinci cases. Most cases were performed by a single console surgeon (69.8%, *n* = 310), while dual console activation ranged from 6.7% to 45.2% across procedures (Table 1). At least one FFB instrument was used in all cases, and 35.8% (*n* = 159) cases were performed using multiple FFB instruments. In order of frequency, Cadiere (66.7%) and Fenestrated Bipolar (32.1%) were the most commonly used FFB instruments, followed by Maryland Bipolar Forceps (25.8%). Needle Drivers were most frequently used for transthoracic esophageal surgery and diaphragm procedures (Table 2). Of all observations, 13% were recorded with FFB set to Off, 21% to low, 42% to medium, and 24% to high. Median console times were lowest for wedge resections and highest for transthoracic esophageal surgery (Table 1).

**Table 1:** Proportion of thoracic procedures performed on the Da Vinci 5 system with force feedback instruments

Procedure	N	Median Console Time IQR)	Dual Console Activation
Anatomic lung resection	258 (58.1%)	95.7 (64.3–141.8) Minutes	74 (28.7%)
*Pulmonary lobectomy*			
*Pulmonary segmentectomy*			
*Wedge to completion lobectomy*			
Wedge Resection	97 (21.8%)	53.4 (37.6–80.6) Minutes	28 (28.9%)
*Pulmonary wedge resection*			
*Pulmonary wedge resection – diagnostic*			
Mediastinal surgery	43 (9.7%)	63.5 (40.5–92.3) Minutes	17 (39.5%)
*Mediastinal mass resection*			
*Thymectomy*			
Transthoracic esophageal surgery	31 (7.0%	216.2 (163.8–327.5) Minutes	14 (45.2%)
*Esophagectomy transthoracic–chest anastomosis*			
*Esophagectomy transthoracic–neck anastomosis*			
*Esophageal diverticulum repair*			
Diaphragm surgery	15 (3.4%)	109.5 (71.7–130.2) Minutes	1 (6.7%)
*Diaphragmatic hernia repair*			
*Diaphragmatic plication*

**Table 2:** Force feedback instruments used by procedure category

Force feedback (FFB) setup	Anatomic lung resection	Wedge resection	Mediastinal surgery	Transthoracic esophageal surgery	Diaphragm surgery
*Number of FFB instruments*					
Single	63.2%	72.2%	65.1%	54.8%	46.7%
Multiple	36.8%	27.8%	34.9%	45.2%	53.3%
*FFB Instrument Type*					
Fenestrated Bipolar	34.1%	34.0%	20.9%	22.6%	40.0%
Cadiere	69.4%	64.9%	74.4%	58.1%	33.3%
Maryland	25.2%	21.6%	34.9%	38.7%	6.7%
Needle Driver	2.7%	0	2.3%	35.5%	66.7%

### Instrument tip forces

**Fig. 1:**
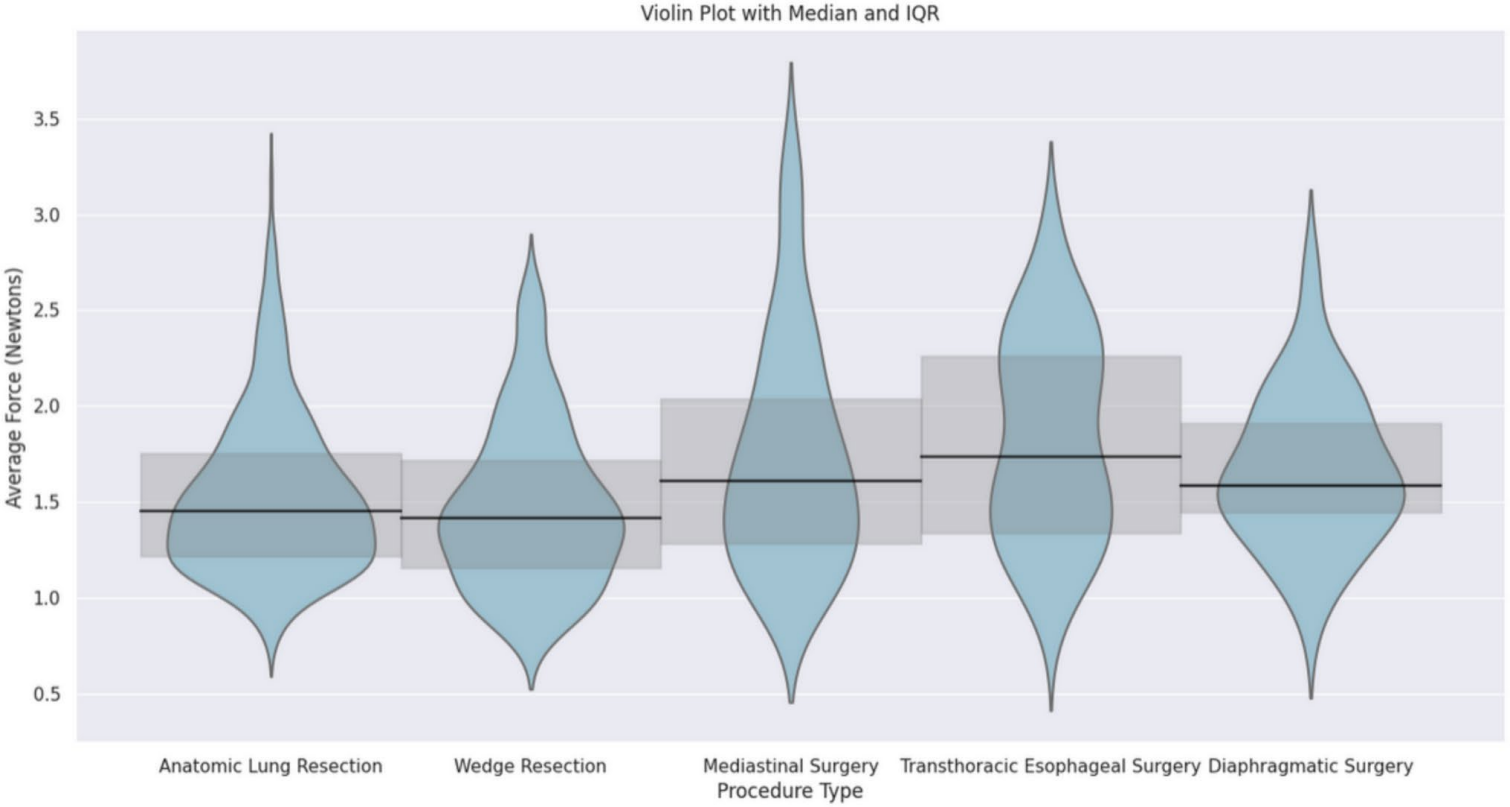
Violin plot of average force applied by the tool tip on the tissue for each procedure in thoracic surgery. Median represented by the horizontal line with 95% confidence interval

The FFB instrument setup used for RATS procedures is summarized in Table 2. The forces experienced at FFB instrument tips tended to be lower for lung resection, anatomic resection 1.45 N (IQR = 1.22–1.75) vs. wedge, 1.42 N (IQR = 1.16–1.72), relative to mediastinal surgery (1.61 N, IQR = 1.28–2.04), esophageal procedures (1.74 N, IQR = 1.34–2.26), or diaphragm surgery (1.59 N, IQR = 1.44–1.91) (Fig. 1). Kruskal–Wallis test revealed significant differences in median force by procedure type (H-statistic = 48.6, *p* < 0.001).

**Fig. 2:**
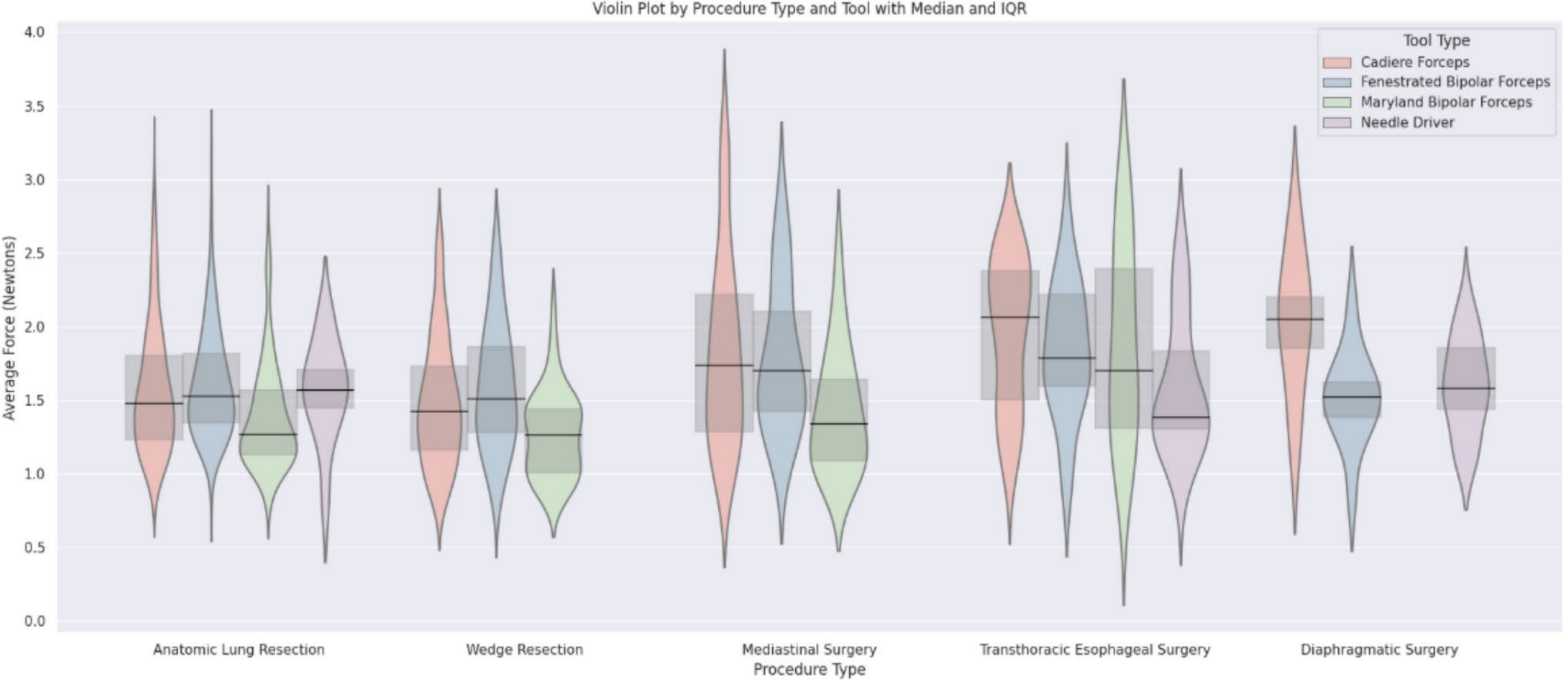
Violin plot of average force applied by the tool tip on the tissue for each procedure by FFB instruments. Forces differed by procedure type and instrument type. Specifically, retraction and suturing instruments had significantly higher median forces than the Maryland Bipolar dissector (H-statistic = 53.4, *p* < 0.001). Median represented by the horizontal line with 95% confidence interval

**Fig. 3:**
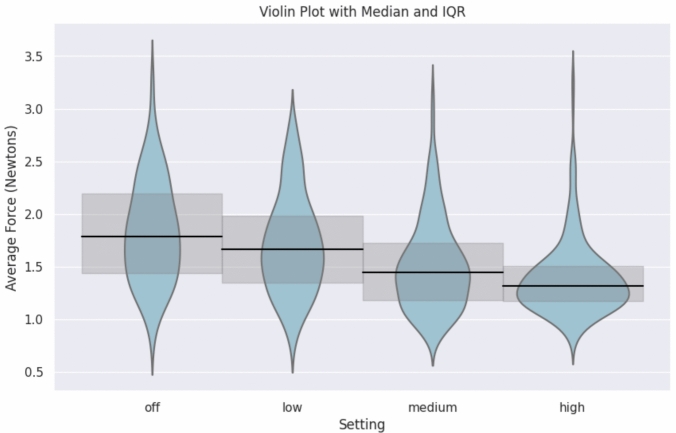
Average force violin plot with median and 95% confidence interval by force sensitivity setting. (H-statistic = 126.6, *p* < 0.001)

**Fig. 4:**
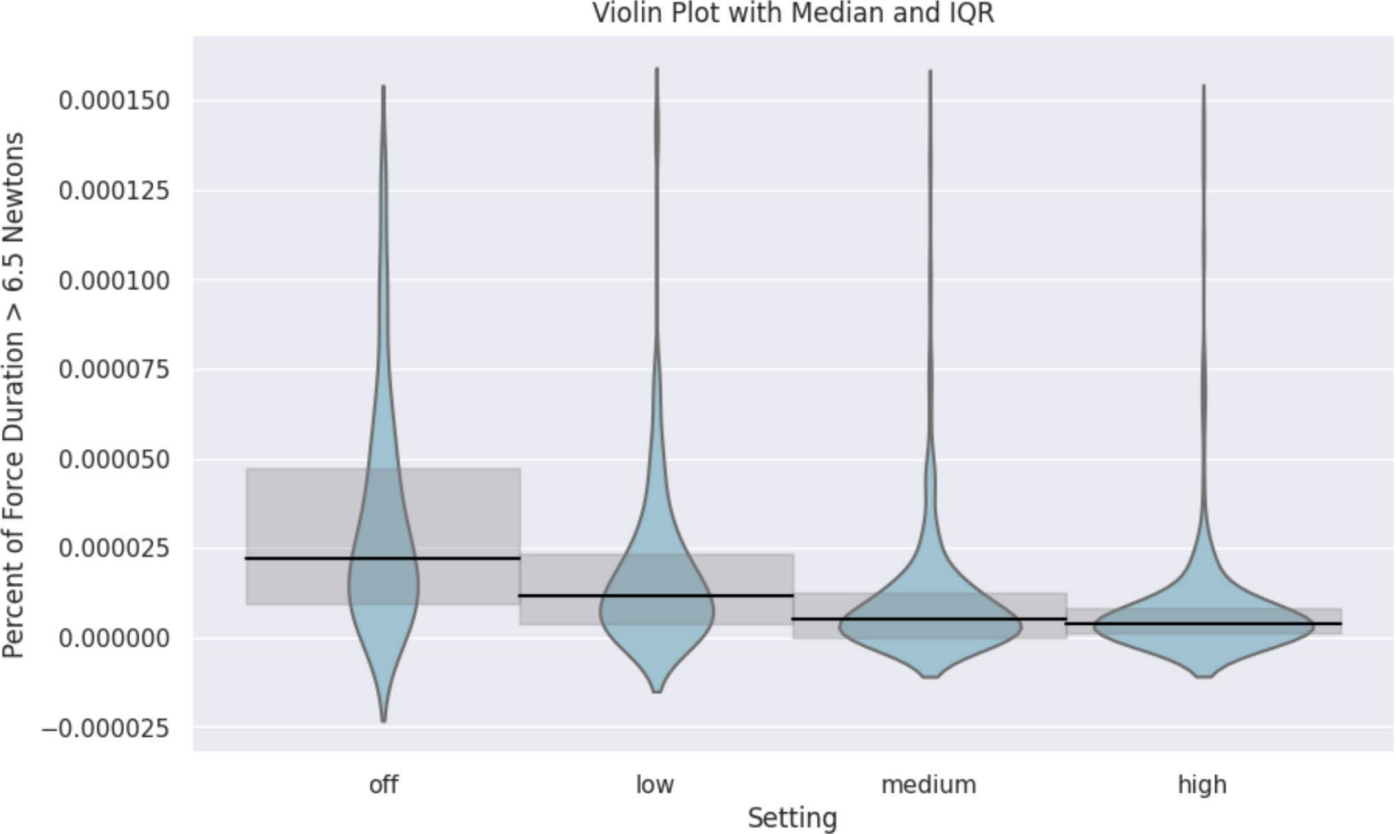
Percent time above 6.5 N violin plot with mean and 95% confidence interval by force sensitivity setting. (H-statistic = 164.8, *p* < 0.001)

Median instrument forces were similarly high between the Fenestrated Bipolar (1.57 N, IQR = 1.34–1.87), the Cadiere (1.50 N, IQR = 1.24–1.91), and the Needle Driver (1.56 N, IQR = 1.31–1.84) and significantly lower for the Maryland Bipolar (1.31 N, IQR = 1.12–1.60, (H-statistic = 53.4, *p* < 0.001). The difference in median forces between instruments for retraction (Cadiere or Fenestrated Bipolar) and suturing (Needle Driver) and dissection instruments (Maryland Bipolar) was observed during anatomic lung resection, wedge resections, and mediastinal surgery, but not during esophagectomy (Fig. 2). Median instrument tip forces decreased stepwise based on the FFB sensitivity setting amongst all procedures: off, 1.79 N (IQR = 1.44–2.19) vs. low, 1.67 N (IQR = 1.35–1.98) vs. medium, 1.45 N (IQR = 1.19–1.73) vs. high, 1.32 N (IQR = 1.17–1.51) (H-statistic 126.6, *p* < 0.001, Fig. 3). The percentage of time at peak force over 6.5 N also decreased significantly for higher FFB settings (off, 0.0022% vs. low, 0.0012% vs. medium, 0.0005% vs. high, 0.0004%; H-statistic 164.8, *p* < 0.001, Fig. 4).

## Discussion

In thoracic surgery, the inability for surgeons to feel force exerted in the operative field has been an important limitation of robotic-assisted approaches. The da Vinci 5 Surgical System, launched in 2024, incorporates FFB, which enables the measurement of force during surgery and allows surgeons to experience haptic sensations. This one-year multicenter experience with FFB technology during robotic thoracic surgery demonstrates that forces experienced at instrument tips varied across different types of thoracic procedures and were highest for retraction instruments. Force feedback instrument use on medium or high settings was associated with surgeons applying less peak force and a reduction in average force by approximately 20%. These findings were seen in cases done by surgeons with substantial experience with thoracic robotic surgery.

Robotic surgery has made substantial progress in minimizing thoracic surgical trauma, and the addition of FFB technology has the potential to further improve patient outcomes, reduce complications, and optimize hospital resource utilization [7]. This study provides clinical evidence that FFB technology may help reduce forces in RATS and result in an objectively gentler surgery. The results build on preclinical evidence that has shown that haptic feedback instruments are effective in reducing instrument force and associated tissue trauma in robotic lung surgery [8–10]. In an ex-vivo study, twenty-eight surgeons at various experience levels, including six thoracic surgeons, performed basic surgical tasks on plant-based surgical simulating organ using the dV5 with FFB instruments [8]. This study demonstrated an average reduction in maximal force of 36% for retraction, 41% for dissection, and 55% for suturing tasks with the use of FFB at the “high” versus “off” sensitivity setting [8]. Experiments using the Saroa surgical system (Riverfield Inc., Tokyo, Japan) with pneumatic force feedback instruments demonstrated that surgeon’s mean grasping force was reduced in the order of 40–50% for interlobar dissection, vascular dissection, bronchial dissection, and lymph node resection on a pig lung model [10]. Another in vivo study in beagle dogs investigated tissue damage from varying grasping forces using the Saroa surgical system [9]. Histological evaluations showed that higher grasping forces exceeding 2 N caused tissue damage in the lung and liver to a higher degree than in other organs [9]. These findings suggest that the haptic feedback instruments could help to reduce surgical trauma in lung surgery. Our results from the first year of experience with FFB in thoracic surgery confirm that the use of FFB instruments on medium- or high-sensitivity settings can maintain the mean instrument forces (plus one standard deviation) below 2 N during RATS procedures. Future research will determine how instrument forces affect clinical outcomes of robotic lung resections, such as chest tube duration, air leak, atrial fibrillation, return of pulmonary function, and patient experience.

FFB technology may aid training in RATS by helping trainees better understand the forces applied to tissues [11]. While robotic instruments offer increased mobility and improved surgical exposure, developing the necessary visual-haptic skills for safe, efficient operating requires time and practice [3, 4]. A multicenter simulation study found that robotic experience level correlated with tissue forces exerted during a suturing exercise on the da Vinci robot, where novice and intermediate level surgeons applied higher maximum and average forces compared to surgeons at the expert level [12]. Force feedback technology has the potential to significantly enhance the learning curve for trainees and early-career surgeons in RATS by restoring a critical sensory input lost from previous robotic platforms. By providing real-time tactile feedback on tissue resistance and instrument forces, this technology enables users to better control the amount of pressure they are applying during delicate maneuvers such as hilar dissection around the pulmonary artery, lymphadenectomy, or bronchial manipulation. Surgeons teaching RATS on the dual console may also be comforted by knowing FFB instruments will limit excessive static force that may not be appreciated visually. Our results show that average instrument forces were highest using retraction instruments such as the Cadiere and Fenestrated Bipolar Forceps, which are typically used on the non-dominant hand (Fig. 2). Activating FFB at higher sensitivity settings on retraction instruments may therefore help handle lung tissues more safely during retraction, potentially lowering the risk of accidental injury and supporting more confident learning. The same applies to surgeons transitioning to robotics. The ability to appreciate the resistance of the anatomical structures may help bridge the gap between open or VATS surgery and RATS. Especially in the early phases of the robotic learning curve, FFB may accelerate skill acquisition and boost confidence.

This study is important because it is one of the first assessments of FFB instruments in thoracic surgery. However, there are several limitations that should be borne in mind. First, its retrospective design introduces the potential for selection bias. The procedures analyzed included some of the first cases performed on the dV5 system, which was launched with highly experienced surgeons. The dual console and FFB instruments were initially not widely available during the initial system launch. Importantly, while the analysis included a large number of cases on a national level, it did not incorporate clinical outcome data, which are necessary to determine the true impact of force feedback on patient safety, recovery, and experience. The > 6.5 N force threshold used to define high force events was selected as the system threshold above which force is not quantified; its clinical significance remains unclear. Additionally, the absence of a control group using a non–force feedback robotic system restricts direct comparisons regarding the FFB technology’s benefit. The choice of FFB instruments and FFB sensitivity settings was not standardized. Differences in instrument force profiles may reflect procedural context or surgeon preference rather than inherent properties of the tools. The average experience level of the 73 participating surgeons with da Vinci surgery was high, which further limits generalizability. The relationship between surgeon experience and instrument force will require future investigation. Finally, the study did not capture subjective surgeon feedback on the usability or perceived value of FFB, which could provide important insights into its role in training and operative performance.

## Conclusions

This study is one of the first to characterize force measured during robotic thoracic surgery and to examine the impact of haptics technology. Forces during thoracic cases varied across procedure and instrument types. Increased FFB settings were associated with surgeons applying less peak force and an approximately 20% reduction in median force during thoracic procedures. Additional research is needed to understand optimal force during surgery and to determine how instrument forces affect clinical outcomes.

## Data Availability

Data are provided within the manuscript.
